# Flowers, leaves or both? How to obtain suitable images for automated plant identification

**DOI:** 10.1186/s13007-019-0462-4

**Published:** 2019-07-23

**Authors:** Michael Rzanny, Patrick Mäder, Alice Deggelmann, Minqian Chen, Jana Wäldchen

**Affiliations:** 1 Department of Biogeochemical Integration, Max Planck Institute for Biogeochemistry, Hans-Knöll-Str. 10, Jena, Germany; 2 Software Engineering for Safety-Critical Systems Group, Technische Universität Ilmenau, Ehrenbergstr. 20, 98693 Ilmenau, Germany

**Keywords:** Species identification, Object classification, Multi-organ plant classification, Convolutional networks, Deep learning, Plant images, Computer vision, Plant observation, Plant determination, Plant leaf, Flower, Poaceae

## Abstract

**Background:**

Deep learning algorithms for automated plant identification need large quantities of precisely labelled images in order to produce reliable classification results. Here, we explore what kind of perspectives and their combinations contain more characteristic information and therefore allow for higher identification accuracy.

**Results:**

We developed an image-capturing scheme to create observations of flowering plants. Each observation comprises five in-situ images of the same individual from predefined perspectives (entire plant, flower frontal- and lateral view, leaf top- and back side view). We collected a completely balanced dataset comprising 100 observations for each of 101 species with an emphasis on groups of conspecific and visually similar species including twelve Poaceae species. We used this dataset to train convolutional neural networks and determine the prediction accuracy for each single perspective and their combinations via score level fusion. Top-1 accuracies ranged between 77% (entire plant) and 97% (fusion of all perspectives) when averaged across species. Flower frontal view achieved the highest accuracy (88%). Fusing flower frontal, flower lateral and leaf top views yields the most reasonable compromise with respect to acquisition effort and accuracy (96%). The perspective achieving the highest accuracy was species dependent.

**Conclusions:**

We argue that image databases of herbaceous plants would benefit from multi organ observations, comprising at least the front and lateral perspective of flowers and the leaf top view.

**Electronic supplementary material:**

The online version of this article (10.1186/s13007-019-0462-4) contains supplementary material, which is available to authorized users.

## Background

The continuing unprecedental loss of species from ecological communities strongly affects properties, functioning and stability of entire ecosystems [1, 2]. Plants form the basis for many terrestrial food webs and changes in plant composition are known to cascade up through the entire community [3, 4], affecting multiple ecosystem functions [5]. Monitoring and managing the presence or abundance of plant species is therefore a key requirement of conservation biology and sustainable development, but depends on expert knowledge in terms of species identification. However, the number of experts can hardly keep pace with the multitude of determination tasks necessary for various monitoring purposes. Automated plant identification is considered to be the key in mitigating the “taxonomic gap” [6, 7] for many professionals such as farmers, foresters or teachers in order to improve neophyte management, weed control or knowledge transfer.

Serious proposals to automate this process have already been published 15 years ago [8] but have only now become an increasingly reliable alternative [9]. Recent boosts in data availability, accompanied by substantial progress in machine learning algorithms, notably convolutional neural networks (CNNs), pushed these approaches to a stage where they are better, faster, cheaper and have the potential to significantly contribute to biodiversity and conservation research [10]. Well trained automated plant identification systems are now considered to be comparable to human experts in labelling plants on images, given the limited amount of information present in the two dimensional images [11].

A considerable hurdle in this research direction has been the acquisition of qualified training images. Today, the ubiquity of smartphones allows people to capture, digitize, and share their observations, providing large quantities of images which may be utilized for the training of classification algorithms. Worldwide citizen science platforms such as Pl@ntNet [7] and iNaturalist [12] show the great potential of crowd-sourcing vast amounts of image data. However, such images inhibit a wide range of quality. A widely known example is the PlantCLEF dataset [13], which is used as benchmark for various computer vision tasks [14–18]. In this collection, each image is assigned a posteriori to one of seven categories (entire, leaf, leaf scan, flower, fruit, stem and branch). Yet, it is not clear how the results achieved on such a dataset are affected by data imbalance towards image number per species and organs, poor image quality and misidentified species [19]. As there is no dedicated sampling protocol for generating these observations, in most cases observations consists of single images [18] of the whole plant or organs taken from undefined perspectives. Other publicly available benchmark datasets such as Oxford flower 102 [20], MK leaf [21] or LeafSnap [22] usually comprise either leaves or flowers but in no case multi organ observations. A recent approach named *WTPlant* uses stacked CNNs to identify plants in natural images [23]. This approach explicitly addresses multiple scales within a single picture and aims at analyzing several regions within the image separately, incorporating a preprocessing step with interactive image segmentation.

Even for experienced botanists it is sometimes impossible to provide a definite identification based on a single image [19], because important details might not be visible in sufficient resolution in order to be recognized and distinguished from similar species. Similar to humans, who increase the chance of correctly identifying plant specimen by observing several organs at the same time, considering more than one viewing angle and taking a closer look at specific organs, combining different perspectives and organs in an automated approach is supposed to increase the accuracy of determination tasks [16, 17]. Especially, separate images of organs and different viewing angles might be beneficial to depict specific small-scaled structures. To our knowledge, the contribution of different perspectives and the fusion of several combinations have never been assessed using a controlled and completely balanced dataset. Therefore, we curated a partly crowd-sourced image dataset, comprising 50,500 images of 101 species. Each individual was photographed from five predefined perspectives, and each species is represented by 100 of those completely balanced observations. The entire dataset was created using the freely available Flora Capture smartphone app [24], which was intentionally developed to routinely collect this type of multi organ observation. We reviewed each image to ensure the quality of species identification and allowing us to address our research questions largely independent of any data quality constraints. More specifically we ask:

- *RQ1* Which are the most and the least important perspectives with respect to prediction accuracy?
- *RQ2* What gain in accuracy can be achieved by combining perspectives?
- *RQ3* How do the accuracies differ among separate CNNs trained per image perspective in contrast to a single CNN trained on all images?
- *RQ4* Is the specificity of a perspective or a combination of perspectives universal or species dependent?
- *RQ5* How sensitive are identification results to the number of utilized training images?

To answer these questions we trained a convolutional neuronal network classifier (CNN) for each perspective and used it to explore the information contained in images from different organs and perspectives.

## Methods

### Image acquisition

**Fig. 1:**
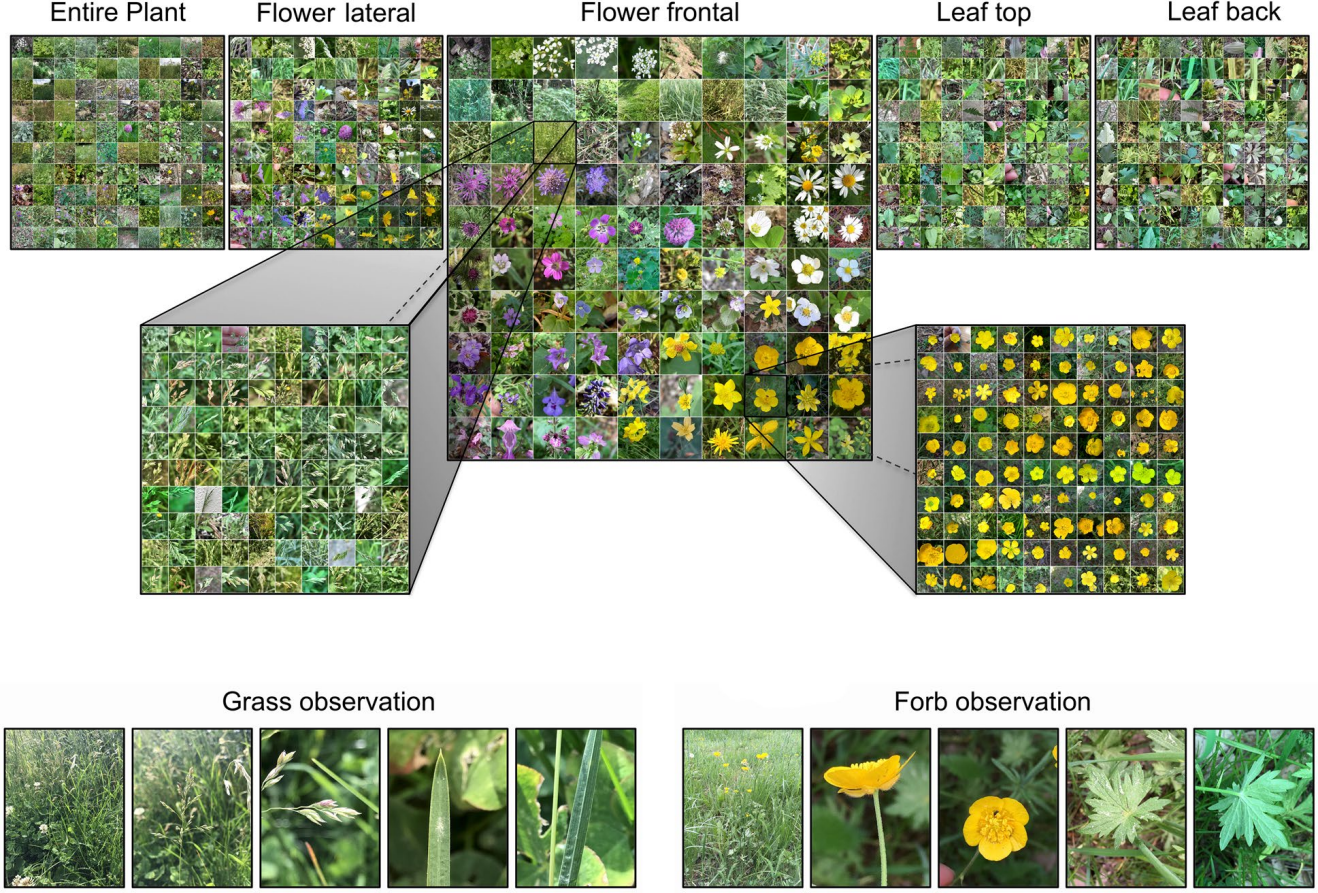
**a** Dataset overview: Each of the 101 species was photographed from five perspectives with 100 repetitions per species. **b** Examples for a complete observation of a grass species (*Poa pratensis*, left) and a forb species (*Ranunculus acris*, right). The perspectives are names entire plant, flower frontal, flower lateral, leaf tip and leaf back. Please note that content definition of the grass perspectives does not match exactly with the definition of the forb species as described in the text. Despite of the slightly different definitions, the perspective names are the same for grass and forb observations. This figure shows only 100 species for the ease of presentation

All images were collected using the Flora Capture app [24], a freely available smartphone application intended to collect structured observations of plants in the field. Each observation is required to consist of at least five images (cp. Fig. 1). For forbs, the following five perspectives are prescribed. First, entire plant—an image capturing the general appearance of the entire plant (referring to the ramet; i.e viable individuals within a clonal colony) taken in front of its natural background. Second, flower frontal—an image of the flower from a frontal perspective with the image plane vertical to the flower axis. Third, flower lateral—an image of the flower from a lateral perspective with the floral axis parallel to the image plane. Fourth, leaf top—an image showing an entire upper surface of a leaf. In the case of compound leafs, all leaflets shall be covered by the image. Fifth, leaf back—the same as before but referring to the leaf lower surface. In the case of composite flowers and flower heads forming a functional unity (i.e., Asteraceae, Dipsacaceae) the flower heads were treated as a single flower. In the case of grasses (Poaceae), this scheme is slightly modified. Instead of the flower frontal view, users are requested to take an image of at least one flower from the minimum focusing distance of their device. The flower lateral view relates always to a side view of the whole inflorescence. Images of entire grass leaves would have too less detail and the image would be dominated by the background. Instead, we requested an image of the upper side of the leaftip (leaf top) and another one taken from the backside in the mid of the leaf (leaf back). These images are also taken at the minimum focusing distance. Despite the slightly different definitions in grass species we always used the names of the forb perspectives for all species. All images are obtained in situ and the users are instructed not to remove any part of the plant while creating the observation. Especially, photos of the leaf backsides required additional manual effort to arrange the objects appropriately and without damage [25]. In the last step, the observations were uploaded to the Flora Incognita server. The correct species for all observations were determined, validated or corrected by the authors of this paper. The citizen science community of the Flora Incognita project [26] was encouraged to particularly contribute observations of species covered by this experiment. Yet, the majority of observations (especially grasses) were obtained by project members and a number of students with a variety of smartphone models, in different regions and with smartphones interchanged among persons. None of the images was preprocessed in any way. The only qualifying condition for an observation was that five images from the predefined perspectives were taken with a smartphone using the Flora Capture App.

### Dataset curation

**Fig. 2:**
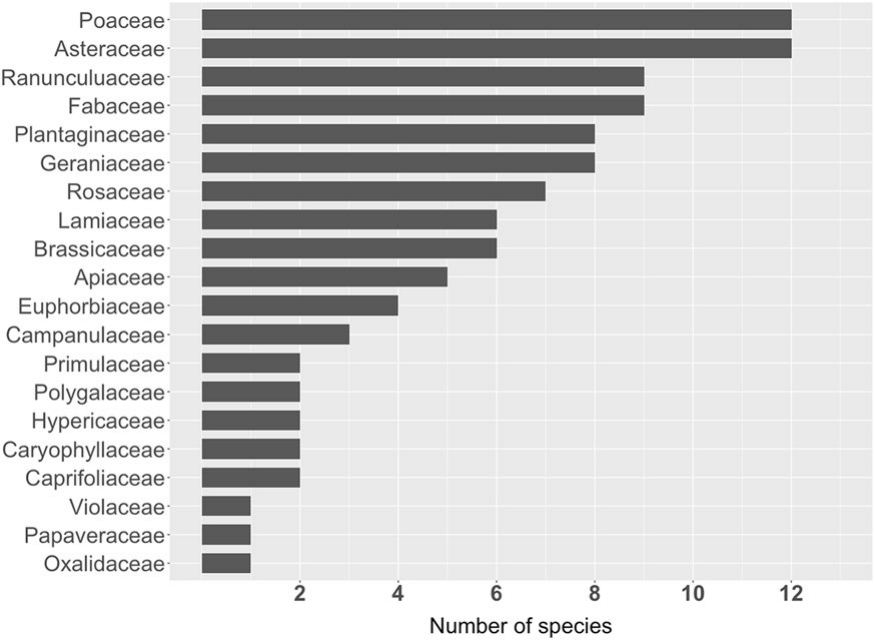
Family membership of the species included in the dataset

The 101 species in the dataset have been selected to mainly represent the large plant families and their widely distributed members across Germany (cp. Fig. 2). Nomenclature follows the GermanSL list [27]. Whenever possible we selected two or more species from the same genus in order to evaluate how well the classifiers are able to discriminate between visually very similar species (see Additional file 1: Table S1 for the complete species list). Each individual was flowering during the time of image acquisition.

### Classifier and evaluation

**Fig. 3:**
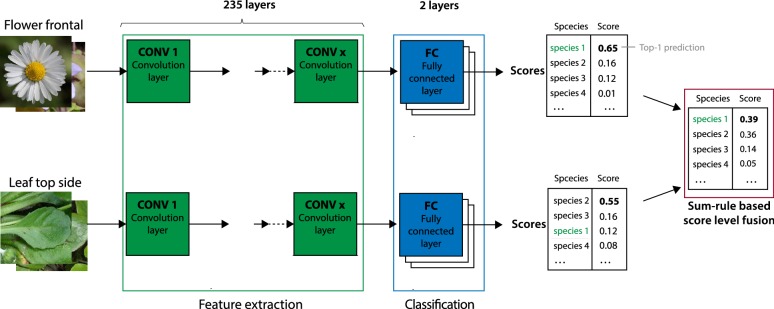
Overview of the approach illustrating the individually trained CNNs and the score fusion of predictions for two perspectives. Each CNN is trained on the subset of images for one perspective, its topology is comprised of 235 convolutional layers followed by two fully connected layers. For each test image the classifier contributes a confidence score for all species. The overall score per species is calculated as the arithmetic mean of the scores for this species across all considered perspectives

We trained convolutional neural network (CNN) classifiers on the described data set. CNNs are a network class applicable to deep learning of images that are comprised of one or more convolutional layers followed by one or more fully connected layers (see Fig. 3). CNNs considerably improve visual classification of botanical data compared to previous methods [28]. The main strength of this technology is its ability to learn discriminant visual features directly from the raw pixels of an image. In this study, we used the state-of-the-art Inception-ResNet-v2 architecture [29]. This architecture achieved remarkable results on different image classification and object detection tasks [30]. We used a transfer learning approach, which is a common and beneficial procedure for training of classifiers with less than one million available training images [31]. That is, we used a network that was pre-trained on the large-scale ImageNet [32] ILSVRC 2012 dataset before our actual training began. Training used a batch size of 32, with a learning rate of 0.003 and was terminated after 200,000 steps. Because an object should be equally recognizable as its mirror image, images were randomly flipped horizontally. Furthermore, brightness was adjusted by a random factor up to 0.125 and also the saturation of the RGB image was adjusted by a random factor up to 0.5. As optimizer for our training algorithms we used RMSProp [33] with a weight decay of 0.00004. Each image was cropped to a centered square containing 87.5% of the original image. Eventually, each image was resized to 299 pixels. We used 80 images per species for training and ten for each validation and testing. The splitting was done based on observations rather than on images, i.e., all images belonging to the same observation were used in the same subset (training, validation or testing). Consequently, the images in the three subsets across all five image types belong to the same plants. We explicitly forced the test set to reflect the same observations across all perspectives, combinations and training data reductions in order to enable comparability of results among these variations. Using images from differing observations in the test, validation and training set for different configurations might have obscured effects and impeded interpretation through the introduction of random fluctuations. In order to investigate the effect of combining different organs and perspectives, we followed two different approaches. On the one hand, we trained one classifier for each of the five perspectives (A) and on the other hand, we trained a classifier on all images irrespective of their designated perspective (B). All subsequent analyses were subjected to the first training strategy (A), while the second one was conducted to compare the results against the baseline approach, as used in established plant identification systems (e.g. Pl@ntNet [7], iNaturalist [12] or Flora Incognita [26]), where a single network is trained on all images. Finally, we applied a sum-rule based score level fusion for the combination of the different perspectives (cp. Fig. 3). We decided to apply a simple sum rule-based fusion to combine the scores of perspectives, as this represents the most comprehensible method and allows a straightforward interpretation of the results. The overall fused score *S* is calculated as the sum of the individual scores for the particular combination as

1 $$\begin{aligned} S = \sum _{1}^{n}\frac{s}{n} \end{aligned}$$

where *n* is the number of perspectives to be fused.

As our dataset is completely balanced we can simply calculate Top-1 and Top-5 accuracy for each species as the average across all images of the test set. Top-1 accuracy is the fraction of test images where the species which achieved the highest score from the classifier is consistent with the ground truth, i.e the predicted species equals the actual species. The Top-5 accuracy refers to the fraction of test images where the actual species is one of the five species achieving the highest score.

### Reducing the number of training images

As the achieved accuracy will be dependent on the number of available training images, we reduced the original number of 80 training images per species to 60, 40 and 20 images. We than repeated the training of CNNs for each of the reduced sets and used each of the new classifiers to identify the identical set of test images. i.e. images belonging to the same ten observations. The difference in accuracy achieved with less training images would indicate whether adding more training images can improve the accuracy of the classifier. On the contrary, if accuracy is unchanged or only slightly lower with the number of training images reduced, this would indicate that adding more training images is unlikely to further improve the results.

## Results

### Performance of perspectives and combinations

**Fig. 4:**
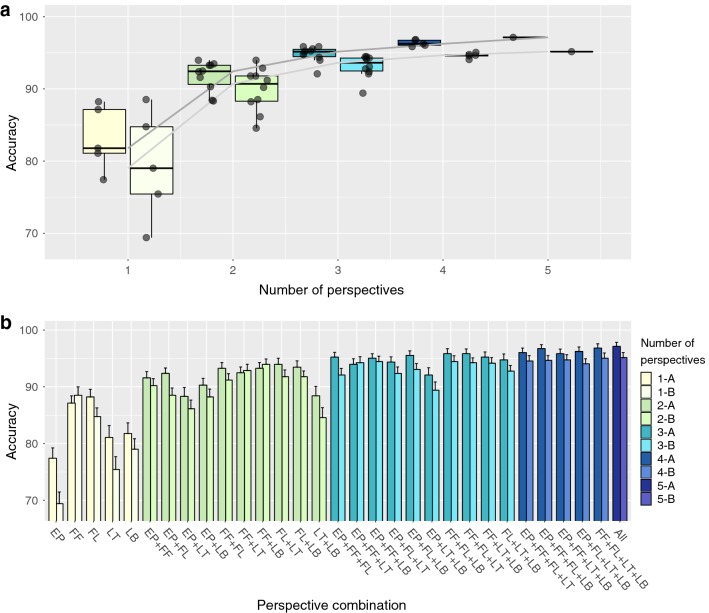
**a** Accuracy as a function of number of combined perspectives. Each data point represents one combination shown in **b**. **b** Mean accuracy for each perspective individually and for all possible combinations. The letters A and B in the legend refer to the different training strategies. The letter A and more saturated colours indicate training with perspective-specific networks while the letter B and less saturated colours represent the accuracies for the same set of test images when a single network was trained on all images. The grey lines connect the medians for the numbers of considered perspectives for each of the training approaches. Error bars refer to the standard error of the mean

Classification accuracy for the single perspectives ranges between 77.4% (entire plant) and 88.2% (flower lateral). Both flower perspectives achieve a higher value than any of the leaf perspectives (cp. Table 1, Fig. 4). Accuracy increases with the number of perspectives fused, while variability within the same level of fused perspectives decreases. The increase in accuracy decreases with every added perspective (Fig. 4) and fusing all five perspectives yields the highest overall accuracy of all combinations (97.1%). The figure also shows that certain combinations with more fused perspectives actually perform worse than combination with less fused perspectives. For example, the accuracy of the best two-perspectives-combination, flower lateral combined with with leaf top (FL + LT: 93.7%), is higher than the accuracy for the worst three-perspective-combination entire plant in combination with leaf top and leaf back (EP + LT + LB: 92.1%).

**Fig. 5:**
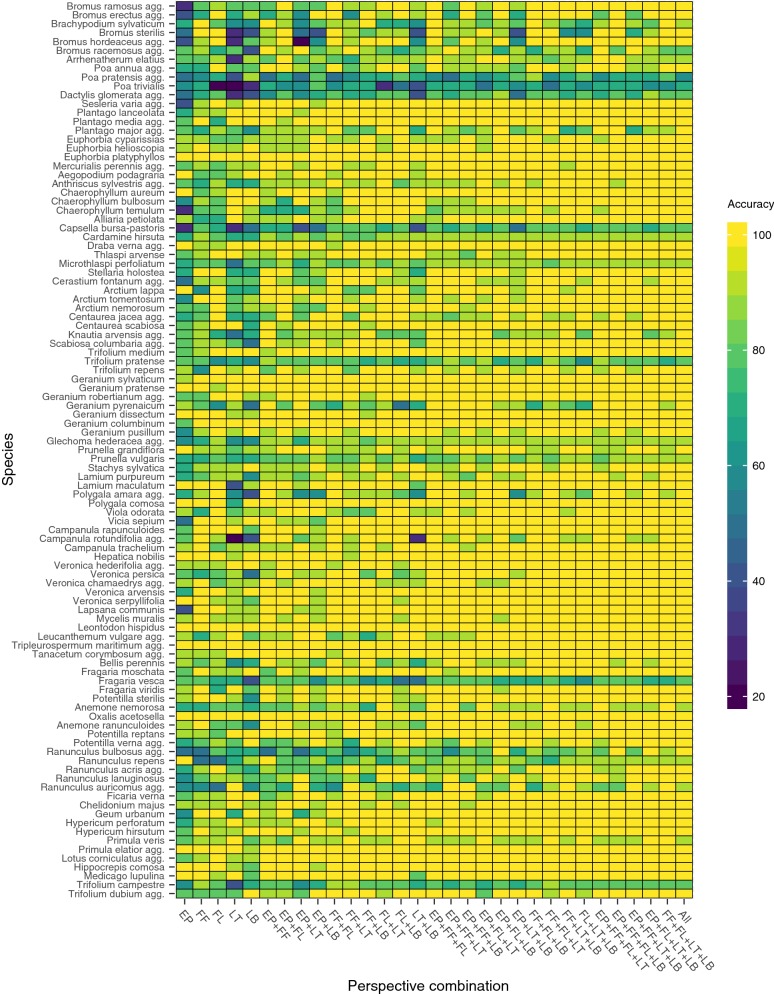
Species wise accuracy for each single perspective and for all combinations of perspectives. Accuracy of a particular perspective combination is color coded for each species

The combination of the two flower perspectives yields similarly high accuracies as the combination of a leaf and a flower perspective, while the combination of both leaf perspectives achieve the second lowest overall accuracy across all two-perspective-combinations with only the combination of entire plant and leaf top slightly worse. The best performing three-perspective combinations are both flower perspectives combined with any of the leaf perspectives. The four-perspectives-combinations generally show low variability and equally or slightly higher accuracies when compared to the three-perspectives-combinations (cp. Table 1, Fig. 4). Fusing all five perspectives achieves the highest accuracy and the complete set of ten images for 83 out of the 101 studied species is correctly classified, while this is the case for only 38 species if considering only the the best performing single perspective flower lateral (cp. Fig. 5).

### Differences among the training approaches

The accuracies gained from the single CNN (approach B) are in the vast majority markedly lower than the accuracies resulted from the perspective-specific CNNs (approach A) (Fig. 4). On average, accuracies achieved with training approach B are reduced by more than two percent compared to training approach A.

### Differences between forbs and grasses

**Fig. 6:**
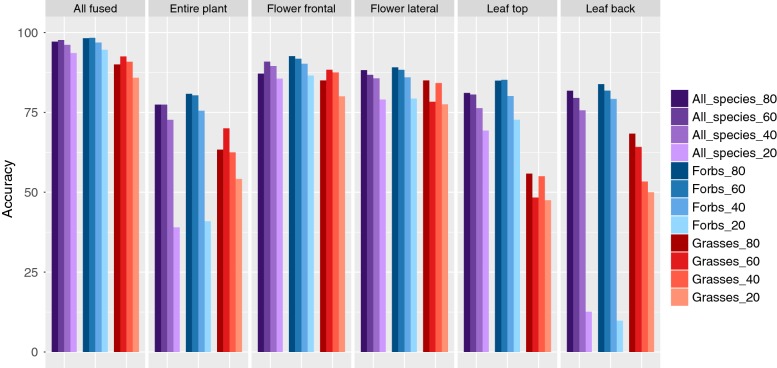
Classification accuracies for the entire dataset (All_spechies), and separately for the subsets grasses and forbs. Numbers next to the dataset in the legend refer to the number of utilized training images

Generally, the accuracies for the twelve grass species are lower for all perspectives than for the 89 forb species (cp. Table 1, Fig. 6). Additionally, all accuracies achieved for the forbs are higher than the average across the entire dataset. Grasses achieve distinctly lower accuracies for the entire plant perspective and for both leaf perspectives. The best single perspective for forbs is flower frontal, achieving 92.6% accuracy alone while the same perspective for grasses achieves only 85.0% (Table 1).

**Table 1:** Top-1 and Top-5 accuracies achieved for each single perspective and for all combinations of perspectives

Analysed	All		Forbs		Grasses
Perspective(s)	Top-1	Top-5	Top-1	Top-5	Top-1
Entire plant (EP)	77.4	92.7	80.8	94.5	63.3
Flower frontal (FF)	87.1	96.5	**92.6**	98.7	**85.0**
Flower lateral (FL)	**88.2**	97.6	89.1	97.9	**85.0**
Leaf top side (LT)	81.1	94.8	84.9	95.6	55.8
Leaf back side (LB)	81.8	95.4	83.8	96.1	68.3
EP + FF	91.6	98.5	94.6	99.1	84.2
EP + FL	92.4	98.4	85.1	96.2	**90.8**
EP + LT	88.3	97.6	91.2	98.1	71.7
EP + LB	90.3	98.2	92.5	98.4	78.3
FF + FL	93.3	98.5	94.0	98.9	87.5
FF + LT	92.5	98.6	**96.4**	99.4	84.2
FF + LB	93.3	98.7	96.1	99.3	88.3
FL + LT	**93.7**	98.7	87.2	96.1	84.2
FL + LB	93.5	99.0	86.7	96.7	85.8
LT + LB	88.4	97.4	91.0	97.8	70.0
EP + FF + FL	95.3	98.9	94.5	98.9	92.5
EP + FF + LT	94.0	99.0	96.5	99.3	82.5
EP + FF + LB	95.1	99.3	96.9	99.6	90.0
EP + FL + LT	94.4	98.9	92.4	98.3	84.2
EP + FL + LB	95.5	99.3	93.3	98.7	84.2
EP + LT + LB	92.1	98.7	94.8	98.8	89.2
FF + FL + LT	**95.8**	99.0	96.8	99.3	87.5
FF + FL + LB	**95.8**	99.1	96.5	99.3	**91.7**
FF + LT + LB	95.3	98.9	**97.4**	99.6	**91.7**
FL + LT + LB	94.8	99.0	92.3	98.0	84.2
EP + FF + FL + LT	96.0	98.9	97.0	99.2	90.0
EP + FF + FL + LB	96.7	99.4	97.4	99.6	**92.5**
EP + FF + LT + LB	95.8	99.3	**97.6**	99.4	86.7
EP + FL + LT + LB	96.2	99.1	95.6	98.9	88.3
FF + FL + LT + LB	**96.8**	99.3	97.6	99.6	90.8
**All**	**97.1**	99.3	**98.2**	99.4	**90.0**

“All” refers to the complete dataset comprising 101 species, “Forbs” refers to the 89 forb species and “Grasses” refers to the twelve Poaceae species. Top-5 accuracy is not shown for “Grasses” due to the low number of species. The highest accuracy per combination level is marked in bold face

### Species-specific accuracy differences

While for some species all test images across all perspectives are correctly identified (e.g., *Oxalis acetosella, Tripleurospermum maritimum*), for other species none of the perspectives or combinations thereof allows the accurate identification of all test observations (e.g., *Poa pratensis, Poa trivialis, Fragaria vesca*). For the majority of species, however, a single or only a few fused perspectives allows a reliable identification. Yet, which kind of perspective achieves the highest accuracy, depends on the species (cp. Fig. 5). For 28 species, none of the single perspective alone allows to identify all test images correctly, while fusing perspectives allows to identify the correct species across all test observations (e.g., *Ranunculus bulbosus, Bromus ramosus, Scabiosa columbaria*).

### Reduction of training images

Reducing the number of training images to 60 or even to 40 images causes no consistent effect on any perspective. Yet, accuracy drops strongly when reducing to 20 training images for the entire plant and leaf back perspectives, while the accuracies for both flower perspectives and the combination of all perspectives are still only slightly affected (Fig. 6).

## Discussion

We found that combining multiple image perspectives depicting the same plant increases the reliability of identifying its species. In general, from all single perspectives entire plant achieved the lowest mean accuracy while the flower lateral perspective achieved the highest accuracies. However, in the specific case the best perspective depends on the particular species. There are several examples where another perspective achieves better results. As a universal best perspective for all species is lacking, generally collecting different views and organs of a plant increases the chance to definitely cover the most important perspective. Especially, images depicting the entire plant inevitably contain lots of background information, which is unrelated to the species itself. In the majority of cases, images of the category entire plant also contain other individuals or parts of other species (Fig. 1a) and it may even be difficult to recognize an individual as a perceivable unit within its habitat. Such background information can be beneficial in some cases, such as tree trunks in the background of typical forest species or bare limestone in the back of limestone grassland species. In other cases, such as pastures, it is hard to recognize a certain focus grass species among others on the image. This similarity in background represents—to a certain degree—a hidden class, which is only partly related to species identity. This could be the reason for the lower accuracies achieved, when a single classifier was trained on all images where much more confounding background information enters the visual space of the network. Visual inspection of test images for species with comparably low accuracy (e.g. *Trifolium campestre* and *Trifolium pratense*) revealed that these contained a relatively higher number of images taken at large distance and were not properly focused. This was possibly due to their small size and low height making it hard for the photographer to acquire proper images.

### Combining perspectives

Flower side view and flower top view provide quite different sources of information which, when used in combination, considerably improve the classification result (Fig. 4). We found that combining perspectives, e.g. flower lateral and leaf top, yields a mean accuracy of about 93.7% and adding flower top adds another two percent, summing to an accuracy of about 95.8% for this dataset. Given that the species in this dataset were chosen with an emphasis on containing congeneric and visually similar species, the accuracies achieved here with a standard CNN setting are considerably higher than comparable previous studies that we are aware of. For example, [18] used comparable methods and achieved an accuracy of 74% for the combination of flower and leaf images using species from the PlantCLEF 2014 dataset. [34] report an accuracy of 82% on the perspectives of leaf and flower (fused via sum rule) for the 50 most frequent species of the PlantCLEF 2015 dataset with at least 50 images per organ per plant. It remains to be investigated whether the balancing of image categories, the balancing of the species itself, species misidentifications or the rather vaguely defined perspectives in image collections such the PlantCLEF datasets are responsible for these substantially lower accuracies. Yet, our results underline that collecting images following a simple but predefined protocol, i.e. structured observations, allows to achieve substantially better results than previous work for a larger dataset and with presumingly more challenging species evaluated with as few as 20 training observations per species.

### Identifying grasses

We are not aware of any study that explicitly addresses the automated identification of grasses (Poaceae). The members of this large family strongly resemble each other and it requires a lot of training and experience for humans to be able to reliably identify these species, especially in the absence of flowers.

While our study demonstrates substantial classification results for most species, the utilized perspectives are not sufficient to reliably identify all species. *Poa trivialis* and *Poa pratensis* are recognized with an accuracy of 60% and 70% respectively, when all perspectives are fused. In vivo, these two species may be distinguished by the shape of the leaf tips and the shape of their ligules. But many of the collected images depict partly desiccated and coiled leaves, which do not reveal those important features. The shape of the ligule, another important character for grass species is not depicted in any of the perspectives used in this experiment. Therefore, we conclude that the chosen perspectives for grasses are still not sufficient to distinguish all species, especially if the identification would only be based on leafs. More research is necessary to identify suitable perspectives allowing to reliably recognize grass species. We assume that the same applies for the related and equally less studied families, such as Cyperaceae and Juncaceae.

### A plea for structured observations

An important obstacle in verifying crowd sourced image data is that in many cases the exact species cannot unambiguously be determined, as particular discriminating characters are not depicted on the image. According to [19], 77.5% of all observations from the first period after launching Pl@ntNet were single image observations and another 15.6% were two image observations leaving less than 7% of all observations to consist of more than two images. Generating multi-image-observations of plants can improve automated plant identification in two ways: (1) facilitating a more confident labelling of the training data, and (2) gaining higher accuracies for the identified species. The more difficult the plant is to identify, i.e. the more help humans need for identification, the more imperative structured observations become. It is important to provide suitable and broadly applicable instructions for users regarding which kind of images are useful to capture in order to gain reliable identification results from automated approach also for challenging species. Applications aiming at automated species identification would benefit from the use of multiple and structured image sets to increase accuracy. Our results suggest that a further increase in accuracy would not necessarily be achieved by acquiring more training data, but rather by increasing the quality of the training data. Generating structured observations are an important step in this direction. However, in contrary to the dataset analyzed in this paper, real world automatic plant identification systems usually suffer from a variety of shortcomings such as them imbalanced distributions, bias between test and training images and unreliable species labels. These shortcomings can be reduced by a huge variety of technical and structural improvements. Nevertheless, encouraging users to provide structured species observations for both training and identification will enhance future improvement of automatic plant identification systems.

## Conclusions

We propose that the recognition rates, especially for inconspicious species and species which are also difficult to differentiate for humans, would greatly benefit from multi organ identification. Moreover, it is even essential to allow for a proper review of such challenging species by human experts, as we found it often impossible to verify the record of a certain species based on a single image. Furthermore, we suggest to improve the observation scheme for grasses and modify our previously utilized perspectives to add further crucial characters such as the ligule. In fact we show that with some constraints on perspective and a thorough review of the images, as few as 40 training observations can be sufficient to achieve recognition rates beyond 95% for a dataset comprising 101 species.

## Additional file

**Additional file 1.** Species list.

## Data Availability

The image datasets used and analyzed during the current study are available from the corresponding author on reasonable request and under copyright restrictions.

## Abbreviations

CNN: convolutional neural network
EP: entire plant
FF: flower frontal
FL: flower lateral
LT: leaf top
LB: leaf back
